# Porcine Acellular Dermal Matrix Increases Fat Survival Rate after Fat Grafting in Nude Mice

**DOI:** 10.1007/s00266-021-02299-z

**Published:** 2021-05-06

**Authors:** Meishu Zhu, Meihui Zhu, Xiaoling Wu, Meiquan Xu, Kunwu Fan, Jinming Wang, Liyong Zhang, Meifang Yin, Jun Wu, Zhixiang Zhu, Guang Yang

**Affiliations:** 1 Department of Burn and Plastic Surgery, Second People’s Hospital of Shenzhen, First Affiliated Hospital of Shenzhen University Health Science Center, Shenzhen, 518000 Guangdong China; 2 Yuanmei Cosmetic and Plastic Medical Center, Shenzhen, China; 3 Department of Pathology, Second People’s Hospital of Shenzhen, First Affiliated Hospital of Shenzhen University Health Science Center, Shenzhen, China; 4 Shenzhen Institute of Translation Medicine, Shenzhen Second People’s Hospital, First Affiliated Hospital of Shenzhen University Health Science Center, Shenzhen, China; 5 Shenzhen Key Laboratory of Health Sciences and Technology, Tsinghua Shenzhen International Graduate School, Tsinghua-Berkeley Shenzhen Institute (TBSI), Tsinghua University, Shenzhen, China

**Keywords:** Porcine ADM, Fat grafting, Extracellular matrix, Liquefaction, Revascularization, Macrophages

## Abstract

**Background:**

Autologous fat grafts have been widely in use for reconstruction, contour abnormalities, and cosmetic surgeries. However, the grafted fat one-year survival rate is unpredictable and always low (20%–80%). Standardizing the existing transplantation technology is difficult due to the limiting conditions. Scaffold materials or drugs are unsuitable to employ because of legal restrictions, complex production, and undetermined hazards. Therefore, a simpler and more effective approach to improve grafted fat survival rate is using commercial products as additives. Earlier studies proved that porcine acellular dermal matrix (PADM), a biomaterial clinically used for wound repair, could work as a scaffold for lipo-implantation. This study aimed at investigating the hitherto unclear effect of PADM on transplanted fat survival.

**Methods:**

Thirty-two 8-week-old female nude mice were divided into two groups. Control mice received a 300 μl fat injection, while the PADM group mice were injected with a 300 μl PADM-fat mixture. After a 4-week treatment, fat weight and liquefaction ratio were assessed. Histological changes were quantified via hematoxylin & eosin (H&E) staining. Macrophage infiltration and vascular regeneration were revealed using an anti-CD34 antibody. Mouse and human mRNA expression levels were gauged via RNA-sequencing. On the third day post implantation, the mRNA expression levels of inflammatory genes *Mcp-1* and *Tnf-α* were measured by qRT-PCR.

**Results:**

The weight of surviving grafted fat did not differ between the control and the PADM group. However, adding PADM significantly decreased fat liquefaction. H&E-stained sections showed that PADM decreased fat necrosis, increased fat tissue regeneration, and raised CD34 levels in the regenerated tissue. RNA-sequencing showed that, compared to controls, fats from PADM-added group expressed more mouse-related mRNA but less human-related mRNA. The following GO and KEGG analysis showed that added PADM increased extracellular matrix (ECM) genes expression levels. The qRT-PCR showed that adding PADM increased *Mcp-1* and *Tnf-α* mRNA expression levels.

**Conclusions:**

In summary, PADM addition increased fat survival rate by reducing fat liquefaction through an increased macrophage infiltration, ECM regeneration, and revascularization. Therefore, PADM addition is a workable application in autologous fat grafting.

**No Level Assigned:**

This journal requires that authors assign a level of evidence to each article. For a full description of these Evidence-Based Medicine ratings, please refer to the Table of Contents or the online Instructions to Authors www.springer.com/00266.

## Introduction

Autologous fat grafting is defined as the transfer of a person’s own fat from one to another body area to enhance or restore volume. The first attempt of autologous fat grafting was made in 1893 [1], and since this procedure has been increasingly used for soft tissue augmentation and reconstruction. However, the grafted fat survival rate was unpredictable. Over the last 100 years, scientists kept perfecting fat treatment technologies and fat grafting techniques to improve the feasibility and longevity of fat grafts. A systemic review [2] revealed that, when a tumescent solution was used, there occurred no significant difference in the results of fat grafts as related to donor sites, harvesting techniques, fat collection cannula sizes, or centrifugal speeds used. Clinical data showed more favorable results with grafted fat processed by centrifugation as compared with sedimentation. In addition, a slower reinjection speed increased the retention rate. In fact, due to remarkable differences in equipment and techniques, several technologies have not been accepted into universal protocols for lipo-implantation. According to recent reports, the one-year fat survival rate ranges still from 20% to 80% [3–5]. The necrotic adipocytes may release compounds inducing inflammation and even multiple complications, such as oil cysts, hematomata, calcifications, or tissue depressions [5–9]. This not only increases the patients’ burden, but also hinders the clinical application of fat grafting.

Besides improving grafted fat survival rate by perfecting clinical methodologies and surgical techniques, another widely accepted method is adding exogenous biomaterials. For example, adipose-derived stem cells (ASCs) have notably been used in plastic surgery, because they have a low immunogenicity, are biocompatible, and are inexpensively and easily obtainable [10]. The disadvantages are also obvious. Due to the nonvascular nature of the transplants, a large proportion of the implanted ASCs will undergo necrosis and be absorbed [11, 12]. Autologous platelet rich plasma (PRP), a known natural reservoir of growth factors, can stimulate tissue repair and regeneration. It is also widely used in autologous fat transplantation to improve fat’s survival rate. A series of studies proved that PRP increased fat survival rate and stem cell differentiation [13–15]. But to extract PRP, it is necessary to collect the patient’s blood. These methods may increase patients’ traumatization. Therefore, it would be preferable to utilize a commercial product to improve grafted fat survival and to avoid more harm to patients.

Porcine acellular dermal matrix (PADM) is a dermal biomaterial that has been stripped of all its cellular elements. As a porcine-derived biological mesh, it is used in the treatment of clinical wound healing has been common. This has been due to PADM’s role in promoting the regeneration of fibroblasts, collagen, and vessels. Eventually, PADM is absorbed and replaced by native tissue. Because PADM is alike human acellular dermal matrix (ADM), it can function as a substitute of human ADM for clinical application. In 2005, Ma et al. [16] proved that PADM can accelerate wound repair in animal experiments. In 2013, Chen et al. [17] found that PADM improved burn wound healing by stimulating collagen synthesis, stem cells proliferation and differentiation, and the expression of relevant growth factors. In 2018, He et al. [18] found that during full thickness cutaneous wound healing, PADM induced M2 macrophage polarization and released a series of wound healing factors, including matrix metalloproteinases (MMPs) and growth factors, which promoted cell proliferation and angiogenesis while remodeling the extracellular matrix (ECM). Although these findings showed that PADM could advance wound healing and tissue regeneration, the effects of PADM in fat grafting have remained unclear. Only few studies showed that PADM may be used as a scaffold for autologous lipo-implantation. Hee et al. [19] found that implanted preadipocytes formed a discrete amount of fat tissue after being cultured in micronized acellular allogenic dermis. Geyol et al. [20] found that adipose stem cells and micronized PADM complexes can be cocultured in vitro. Later transplantation experiments confirmed that cultured adipose stem cells can still differentiate into adipocytes. These earlier studies showed that PADM can be used as a safe injectable three-dimensional soft tissue filler. However, hitherto studies are lacking that would confirm a beneficial role of PADM in grafted fat survival.

We posited that PADM could increase grafted fat survival by promoting vascular and tissue regeneration. Therefore, we analyzed fat grafts success rate in relation to three distinct aspects: fat survival rate, histology changes, and RNA expression levels.

## Materials and Methods

### Fat Preparation

We performed all procedures and protocols according to the recommendations of the National Institutes of Health Guidelines as approved by Shenzhen Second People’s Hospital (KS20190712004). A 35-year-old female voluntarily donated fat and signed the informed consent. After tumescent local anesthesia, fat was taken from the back of the thigh. The collected fat was stored in 50 ml syringes and was washed in buffer (0.9% normal saline 500 ml, insulin 30 U) thrice until its color turned golden. 0.2 g PADM particles (#6846, Unitrump Bio, Qidong, China) with a diameter of 0.5 mm were mixed with 20 ml of washed fat inside a 50 ml syringe. The PADM-fat mixture was aliquoted into 1 ml syringes with a blunt needle and injected at devised sites.

## Animals

We performed all experimental procedures and protocols following the recommendations of the National Institutes of Health Guidelines for the Care and Use of Laboratory Animals as approved by the Institutional Animal Care and Use Committee of Shenzhen Second People’s Hospital (KS20190712004). Protocols did abide by all relevant guidelines and laws. Thirty-two 8-week-old female BALB/c background nude mice were bought from Guangdong Medical Laboratory Animal Center (Guangzhou, China). Mice were fed a standard chow (1025; HFK, Guangzhou, China) and tap water ad libitum. Lighting cycles were 12 hours of illumination and 12 hours of darkness. Four to five mice in a cage. The temperature was around 20 °C–24 °C. Humidity was about 50-55%. Mice were euthanized with CO2.

**Fig. 1:**
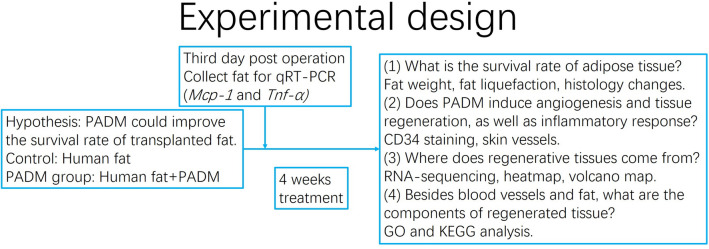
Experimental design

Mice were separated into two groups. After isoflurane (970-00026-00, RWD, Shenzhen, China) anesthesia, the control group and the PADM group mice were subcutaneously injected in the back with 300 μl fat and 300 μl PADM-fat mixture, respectively. Mice were sacrificed 4 weeks later. Fat samples were collected and preserved for further analyses. The experimental design is shown in Fig. 1.

## Fat Measurement

The fat survival rate was assessed via two criteria, i.e., surviving fat weight and fat liquefaction ratio. Fat and PADM-fat mixture weights were measured using electronic scales. Surviving fat weight (%) was equal to surviving fat weight divided by fat weight before the injection. Fat photos were captured with cell phone camera (Mate 9 pro, Huawei, Shenzhen, China). Fat liquefaction ratio was obtained by measuring the fat weight after separation of the liquefied fat. The fat was placed on a filter paper and pressed. The liquefied fat was absorbed by the filter paper, and the remaining fat was weighed. Fat liquefaction ratio (%) was equal to (surviving fat weight minus fat weight after oil removal) divided by surviving fat weight. Fat with abnormal color was excluded from the results.

## Histology

Fat samples were preserved in 4% paraformaldehyde (PFA) overnight. Samples were routinely embedded in paraffin and cut into 5 μm thick sections. Slides were stained with H&E (PH1732, Phygene, Fuzhou, China) and anti-CD34 antibody (HA-ab-ET1606-11, HUABIO, Hangzhou, China). Pictures were captured with a Zeiss microscope (Axiocam 503, Zeiss, Jena, Germany) using Zen lite software (Version 2.1, Zeiss, Jena, Germany). Pictures were analyzed with ImageJ (https://imagej.nih.gov/ij/). The stained parts were selected and their percentage area was measured.

The H&E staining procedure was performed according to manufacturer’s instructions. For CD34 staining, the slides were incubated at 65 °C for 30 min, dewaxed with alcohols and kept in water. Slides were soaked in a pH 6.0 citrate solution at 95 °C for 15 min, 3% H2O2 for 10 min, anti-CD34 antibody for 60 min, and horseradish peroxidase (HRP) secondary antibody (111-035-144, Jackson ImmunoReasearch, West Grove, USA) for 15 min. Diaminobezidin (DAB, DAB-1031, Maixin, Fuzhou, China) staining lasted for 5 min, and hematoxylin staining for 15 seconds. Slides were sealed with neutral resin.

## Microvascular Regeneration

Dorsal skins connected to the fat samples were preserved in PBS. Photos were captured with microscope and camera (NSZ608T&DC6000, Jiangnan Yongxin, Ningbo, China). When the blood vessels thicken or microvasculars appear, it is considered that there is obvious vascular regeneration.

## RNA Sequencing

Four weeks after treatments, fat samples were preserved in RNA Later at 4 °C overnight. On the next day, samples were stored in dry ice and sent to the RNA-sequencing company (Majorbio, Shanghai, China). The data were processed by cluster heatmap, Gene Ontology (GO), and Kyoto Encyclopedia of Genes and Genomes (KEGG) enrichment analysis through the Majorbio cloud platform (www.majorbio.com).

## Quantitative Real-Time (qRT)-PCR

Fat samples were preserved in RNA Later (AM7020, Invitrogen, Carlsbad CA, USA) at 4 °C overnight. Total RNA was isolated using TRIzol® reagent (9109, Takara, Kyoto, Japan). The cDNAs were synthesized using PrimeScript RT reagent Kit (RR047A, Takara, Kyoto, Japan). The qRT-PCR was performed in an ABI-7300 (ABI, Foster California, USA) using SYBR Green (B21203, Bimake, Shanghai, China) according to manufacturer’s instructions [21]. Macrophage biomarkers were chosen according to the earlier literature [22]. Primers: monocyte chemotactic protein-1 (*Mcp-1*), forward 5'-CCAGCCTACTCATTGGGATCA-3', reverse5'-CTTCTGGGCCTGCTGTTCA-3'; tumor necrosis factor-alpha (*Tnf-α*),forward, 5'-ACGTCGTAGCAAACCACCAA-3', reverse, 5'-GCAGCCTTGTCCCTTGAAGA-3'; collagen type XII alpha 1 (*Col12a1*), forward 5'-AGGCAGAAGTTGACCCACCT-3', reverse 5'-CAGTGGTACTAGCTGCAAGGG-3'; collagen type VIII alpha 2 (*Col8a2*), forward 5'-TGCCCCGGTAAAGTATGTGC-3', reverse 5'-GCATCGGTAGAGGCATTTCCA-3'; vascular endothelial growth factor A (*Vegfa*), forward 5'-CTGCTGTAACGATGAAGCCCTG-3', reverse 5'-GCTGTAGGAAGCTCATCTCTCC-3'; epidermal growth factor-containing fibulin-like extracellular matrix protein 1 (*Efemp1*), forward 5'-AGTGTGCAGCAGGCTATGAAC-3', reverse 5'-TTTGGTGGCAATATGGAGGCA-3'; fibroblast growth factor 18 (*Fgf18*), forward 5'-CTGCGCTTGTACCAGCTCTAT-3', reverse 5'-GACTCCCGAAGGTATCTGTCT-3'; chemokine (C-X-C motif) ligand 5 (*Cxcl5*), forward 5'-GTTCCATCTCGCCATTCATGC-3', reverse 5'-GCGGCTATGACTGAGGAAGG-3'; glyceraldehyde-3-phosphate dehydrogenase (*Gapdh*), forward,5'-AGGTCGGTGTGAACGGATTTG-3',reverse 5'-GGGGTCGTTGATGGCAACA-3'. *Gapdh is* the housekeeping gene.

## Statistics

Statistical analyses were conducted using GraphPad Prism software (V6.0, San Diego, CA, USA). Data were presented as means ± standard deviations (SDs). Two-tailed Students' t test served to evaluate the significance of the differences between the two experimental groups. A probability value (*P*) of was less than 0.05 was considered as significant. *P<0.05; **P<0.01; ***P<0.001; ****P<0.0001.

## Results

### PADM Increased Grafted Fat Survival Rate by Decreasing Fat Liquefaction

**Fig. 2:**
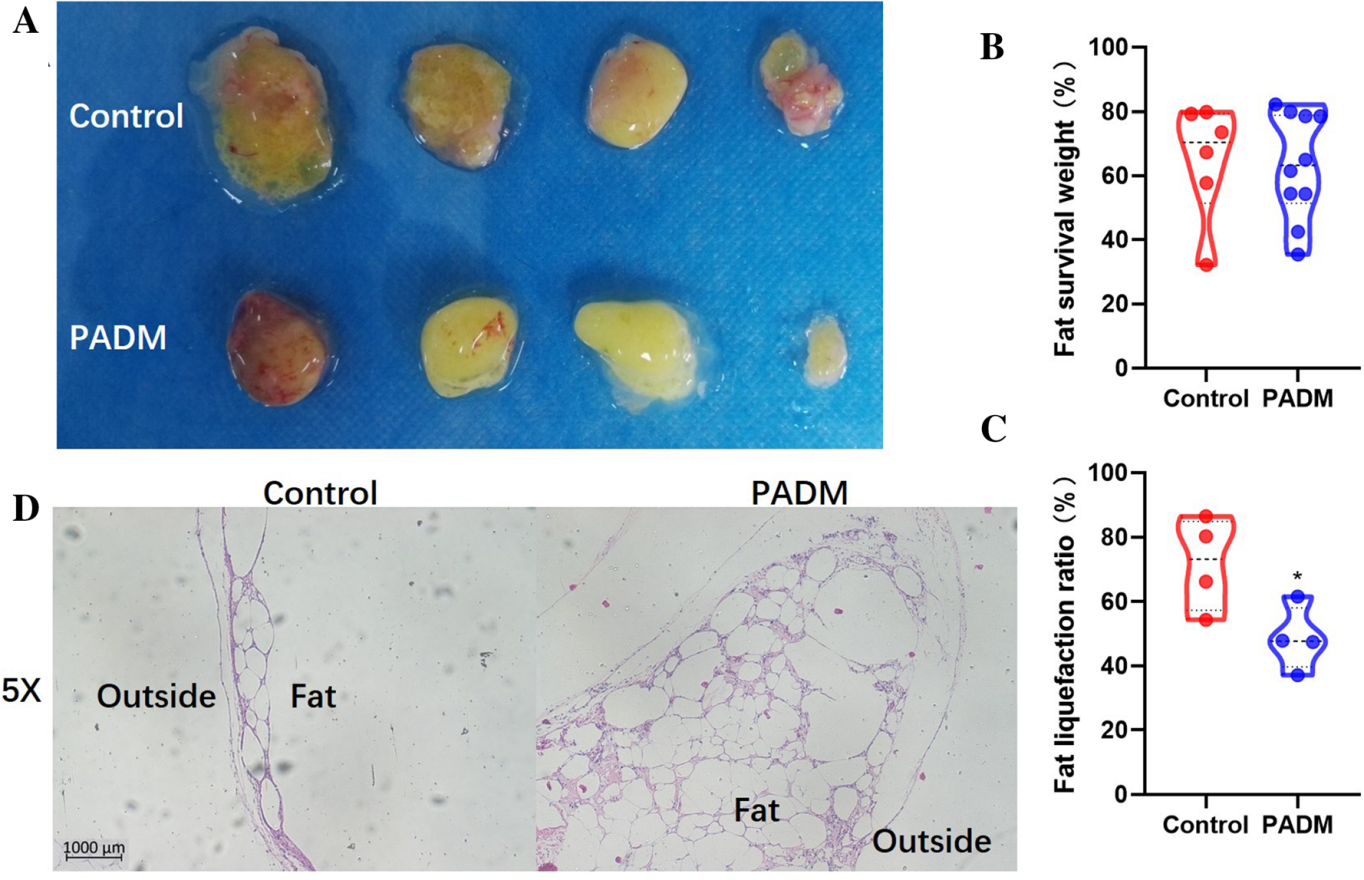
Fat survival rate in mice experiment. **a** Representative pictures of fat chunks 4 weeks after transplantation. **b** The surviving fat weight % was alike in the control and PADM group (controls, 64.96 ± 18.05 vs. PADM, 63.22 ± 16.57 %, *P* = 0.8463, n = 6−10). **c** PADM addition significantly reduced the fat liquefaction ratio % (controls, 71.82 ± 14.39 vs. PADM, 48.47 ± 10.03 %, *P* = 0.0374, *n* = 4). **d** H&E-stained fat tissues. The left panel shows a typical section from the control mice group. The right panel shows a typical section from the PADM mice group. *, *P*<0.05

To evaluate PADM’s effects, fat survival rate was first measured. Compared to the control group, PADM did not change surviving fat weight (Fig. 2a, b), but significantly reduced fat liquefaction ratio (Fig. 2c). To further confirm the effects of added PADM on fat liquefaction, the changes in tissue histology were analyzed in stained slides. In the control group, only one layer of fat particles was seen at the edge of the transplanted fat and it was rare to see traces of H&E staining in the central area (Fig. 2d, left panel). In the added PADM group, fat particles were evenly distributed in the fat and some areas had taken the H&E stain (Fig. 2d, right panel). Thus, the results showed that PADM reduced adipocyte necrosis in the fat central area while inducing tissue regeneration.

## PADM Increased Vascular and Tissue Regeneration

**Fig. 3:**
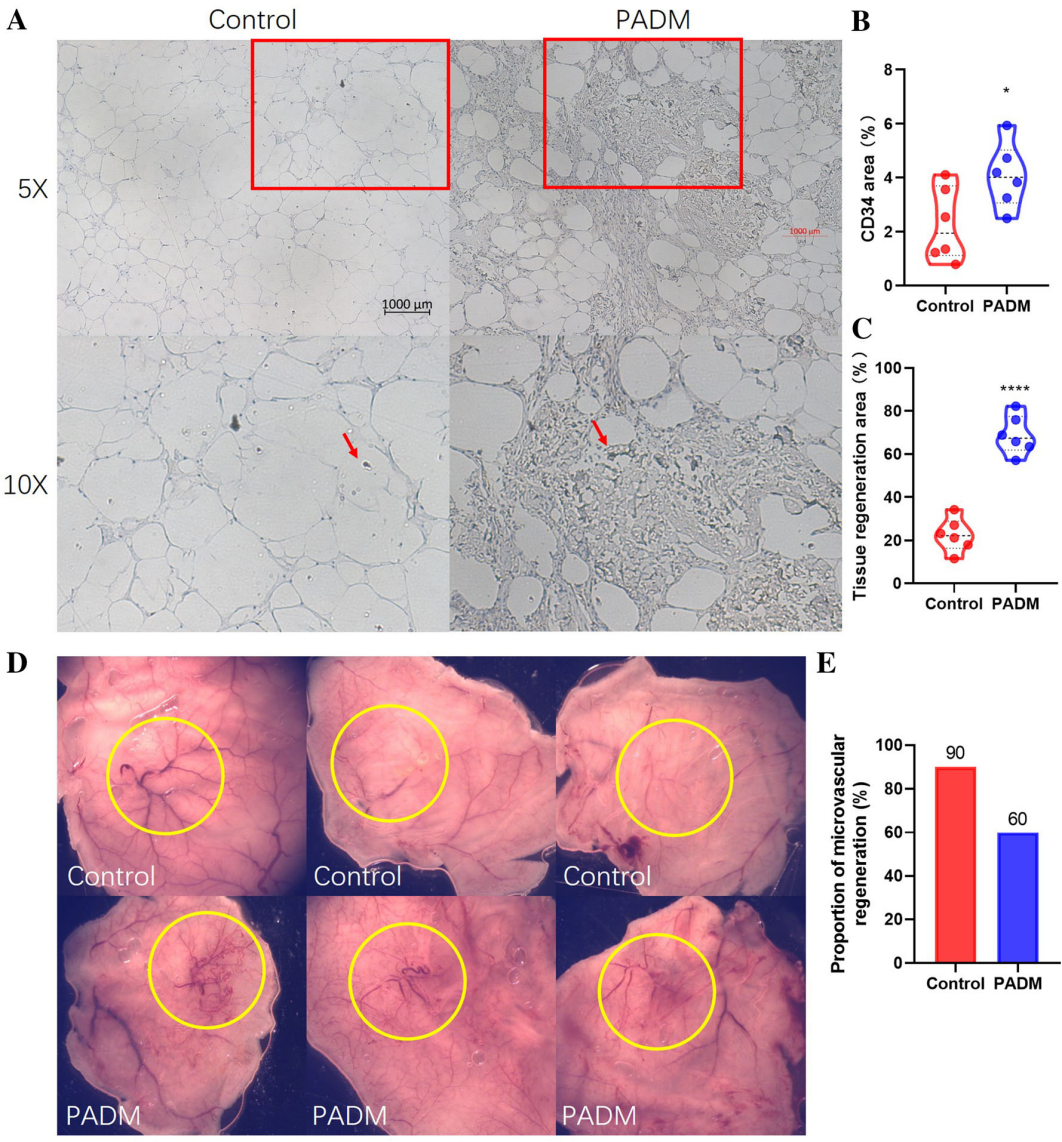
Vascular and tissue regeneration. **a** Representative pictures of fat slides. The red arrows point to the CD34 color. **b** CD34-positive area statistics (controls, 2.264 ± 1.357 vs. PADM, 4.071 ± 1.195 %; *P* = 0.0345; n = 6). **c** Tissue regeneration area (controls, 22.54 ± 7.782 vs. PADM, 68.89 ± 9.012 %; *P* <0.0001, *n* = 6). **d** Representative pictures of dorsal skin. The yellow circles point to the microvascular regeneration area. **e** The proportion of mice with significant regeneration of microvascular (controls, 60% vs. PADM, 90%; *n* = 10). *, *P *< 0.05. ****, *P* < 0.0001

Vascular regeneration is crucial to tissue regeneration. To reveal the vascular regeneration in the surviving fat, the slides were stained with a mouse anti-CD34 antibody, a biomarker of vascular cells and macrophages. Compared to the control group, PADM addition not only increased the fat tissue regeneration, but also significantly raised CD34’s expression levels in the regenerated fat tissue (Fig. 3a, b, c). While human anti-CD34 antibody failed to stain the slides (data not shown). To reveal the vascular regeneration in the connected tissues, the dorsal skins were collected and observed. Compared to the control group, PADM addition increased the microvascular regeneration proportion (Fig. 3d, e). Therefore, adding PADM increased inflammatory cells infiltration and vascular regeneration.

## PADM-Induced Tissue Regeneration Is Mice-Related

**Fig. 4:**
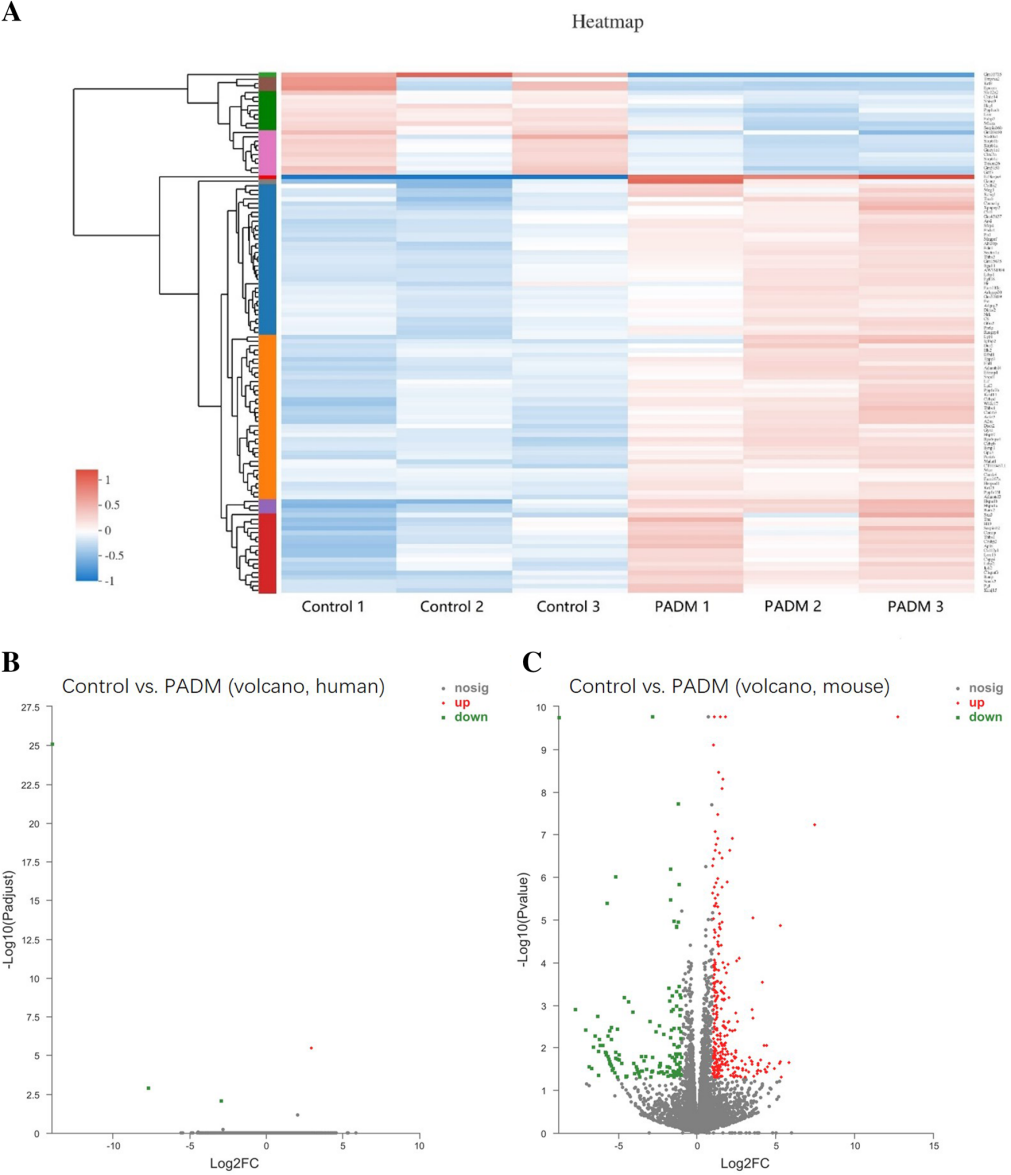
RNA-sequencing. **a** RNA cluster analysis. The difference of RNA expression (Volcano map) of human **b** or mice **c** between the control group and PADM group genes. *N* = 3

Because the tissue can be stained by mouse CD34 antibody, not by human CD34 antibody. To understand where the regenerative tissue came from, we compared the differences in gene expression levels between the two groups. The cluster analysis of RNA sequencing showed that the expression levels of 117 RNAs of the PADM-added group significantly differed from the control group. The expression levels of the three samples belonging to the control group were similar, as were those of the three samples belonging to the experimental group (Fig. 4a). The expressed genes were related to those of humans and mice. The Volcano analysis showed that there occurred significant differences in the mouse genes expression levels (Fig. 4c), but not in human ones (Fig. 4b). This result proves that the regenerated tissue derived from mice cells.

**Fig. 5:**
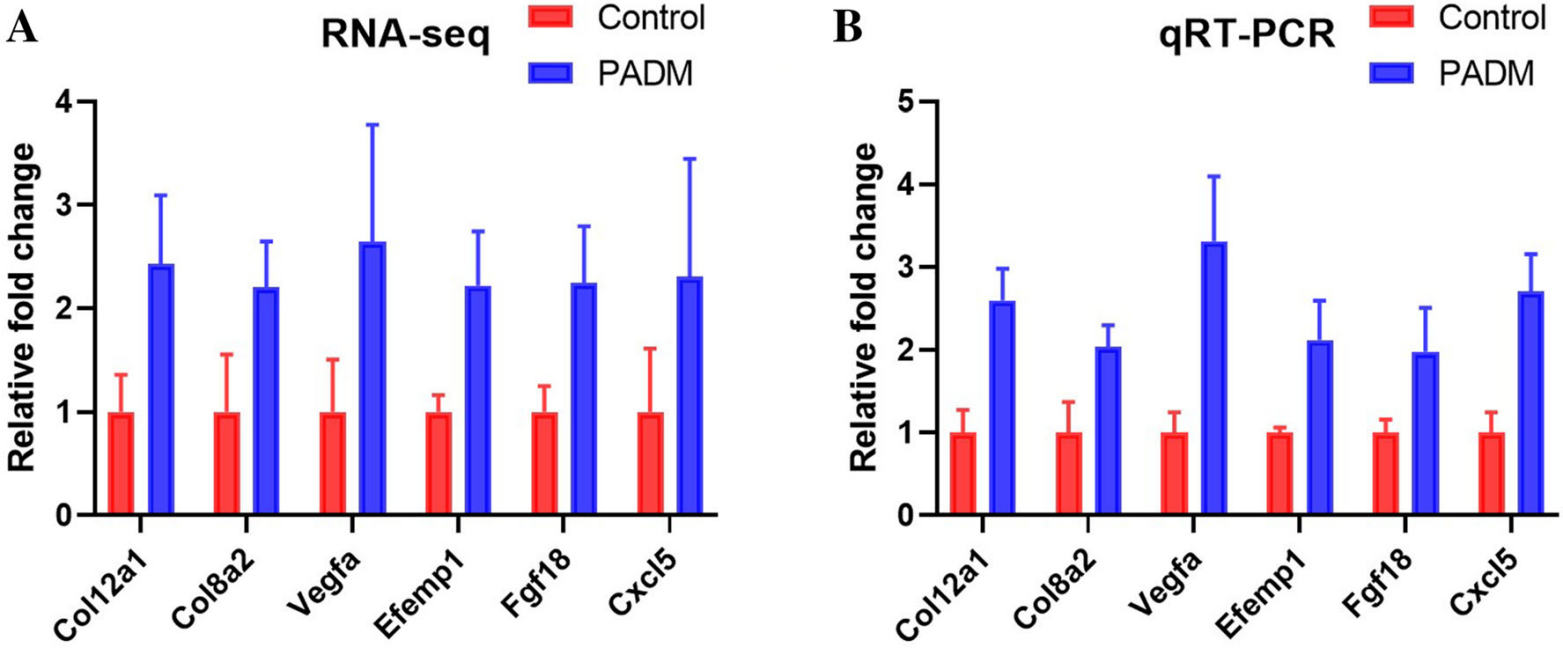
The validation of the expression of genes by qRT-PCR. The original data from RNA-sequencing were presented in A and qRT-PCR results in B. *N* = 3

To confirm the RNA-sequencing data, six differentially expressed genes (*Col12a1*, *Col8a2*, *Vegfa*, *Efemp1*, *Fgf18*, *Cxcl5*) were selected for validation by qRT-PCR. The original data from RNA-sequencing are presented in Fig. 5a, and qRT-PCR results are shown in Fig. 5b and results of qRT-PCR revealed a similar expression pattern as the original RNA-sequencing data, which indicated the correctness of gene expression analysis.

## PADM-Induced Tissue Regeneration Is Related to ECM

**Fig. 6:**
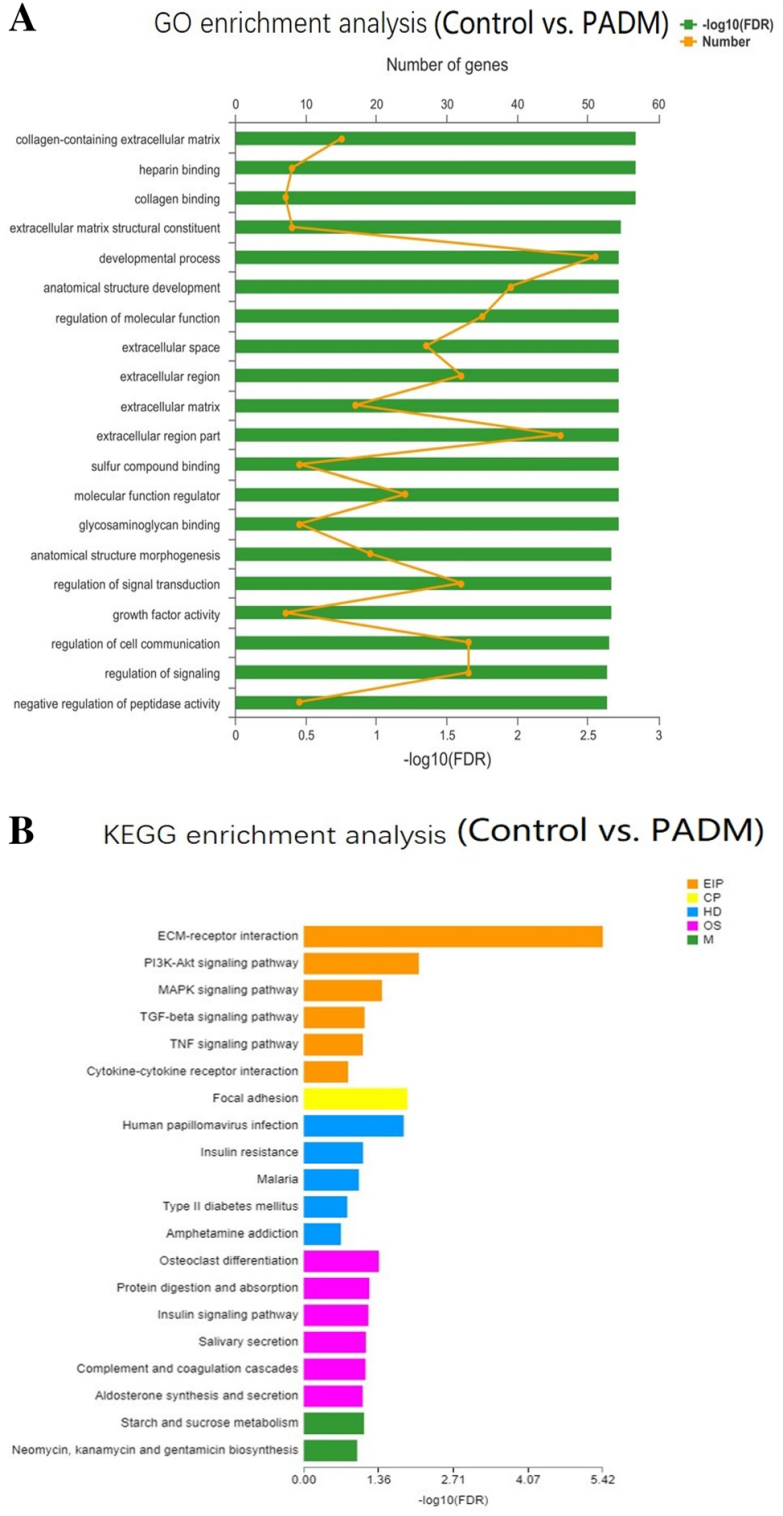
GO and KEGG enrichment analysis. **a** GO enrichment analysis between the control and PADM group. **b** KEGG enrichment analysis between the control and PADM-added groups. *N* = 3

To investigate the biological process of PADM-induced tissue regeneration, we further analyzed the RNA-sequencing results. The GO analysis showed that PADM addition influenced the RNA expression of genes related to collagen, ECM, anatomical structure, and growth factor activity (Fig. 6 a). We also used KEGG to analyze RNA expression. The results showed that PADM’s effects on RNA expression involved ECM-receptor interactions, cytokine-cytokine receptor interactions, and the signaling pathways of phosphatidylinositol-3-kinase (PI3K)-Akt, mitogen-activated protein kinase (MAPK), transforming growth factor (TGF)-β, and tumor necrosis factor (TNF)-a (Fig. 6b). This result proves that the regenerated tissue is related to ECM.

## PADM Increased Inflammatory Responses on the Third Day Post Implantation

**Fig. 7:**
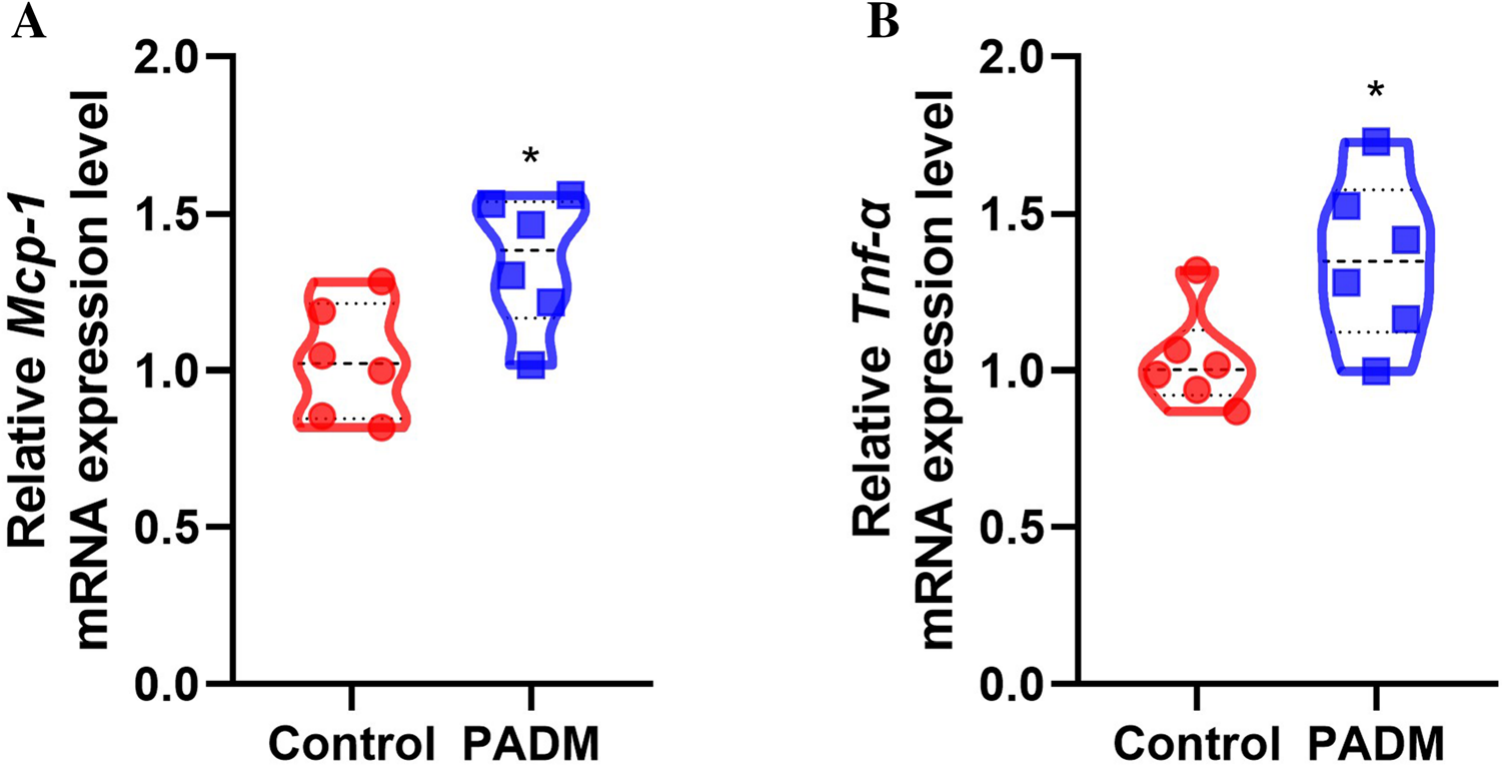
Expression levels of proinflammatory factors at day 3 post transplantation. **a** *Mcp-1* expression levels (controls, 1.032 ± 0.1823 vs. PADM, 1.349 ± 0.2098 %; *P* < 0.05; *n* = 6). **b** *Tnf-α* expression levels (controls, 1.033 ± 0.1556 vs. PADM, 1.352 ± 0.2626 %; *P* < 0.05; *n* = 6). *, *P* < 0.05

To investigate whether PADM-induced macrophage infiltration is related to the expression of proinflammatory genes in the early stage of transplantation, fat samples were isolated on the third day after transplantation. The qRT-PCR results showed that, compared to the control group, PADM significantly increased *Mcp-1* and *Tnf-α* mRNA expression levels (Fig. 7).

## Discussion

It is always clinically difficult to improve the fat survival rate after fat grafting. In the last 30 years, PADM has been widely used in clinical tissue repair, especially in the field of dermal tissue replacement and tissue regeneration scaffolds [23]. Here, we prove for the first time that PADM can also be used in the field of fat grafting as it improves the grafted fat survival rate. PADM addition appears to promote angiogenesis by activating macrophages, so that nutrients can be transported into the grafted adipose tissue promoting ECM regeneration, and ultimately reducing fat liquefaction and adipocyte necrosis. Our results add a new convenient and safe material and technology to cosmetic surgery.

Although the fat survival rate can be improved by perfecting the fat treatment method and reducing the reinjection speed, these protocols are hard to standardize because of limitations of equipment and operative skill. Using stem cells as additives is also a valid method to improve the fat survival rate. Yet, the clinical application of stem cells is still subject to legal restrictions. Adding growth factors to promote adipose tissue regeneration is also an effective method. However, it usually takes a long time to adequately evaluate the safety of drugs translated from research & design to application, and the costs are exceedingly high. PRP is also an ideal additive in fat grafting. But the extraction of PRP is an invasive method. PADM, as a widely used product in clinical settings for many years, has proved to be very safe. Moreover, PADM has several advantages, such as low immunogenicity, commercialization, and low price. Our study also shows that it is easy to prepare and inject the PADM-fat mixture, which beneficially improves the fat survival rate. Therefore, PADM features are conducive to its clinical application.

Fat liquefaction is one of the main reasons of fat grafting failure [24]. This is usually caused by an insufficient blood supply of the fat tissue after operation, resulting in its aseptic necrosis, which by releasing more oozing fluids negatively affects postoperative healing [25]. Here, we found that adding PADM significantly reduced fat liquefaction and improved fat survival. To figure out whether the decrease in fat liquefaction is related to the increase in blood supply, a histological study was performed on surviving fat samples. The results showed that added PADM significantly increased CD34 expression in fat mass by promoting the infiltration of inflammatory cells and the regeneration of blood vessels. To further confirm the existence of an inflammatory response, we also studied the mRNA expression of *Mcp-1* and *Tnf-α* genes on the third day post operation. Compared with the control group, the expression of these proinflammatory factors in the adipose tissue of the PADM-added group was significantly upregulated. This finding suggests that angiogenesis may be related to an early inflammatory cell infiltration following surgery. Cai et al. [26] found that the early depletion of macrophages reduced angiogenesis, weakened the recruitment of Sca-1+/CD45+ stem cells, and ultimately led to reduced fat survival. On the contrary, the early activation and polarization of macrophages (due to TGF-β’s high expression) promoted the blood-derived stem cells infiltration, showing that macrophages were essential for tissue revascularization. Consistent with these results, we confirmed that PADM does improve fat survival rate and reduces fat liquefaction by increasing inflammatory cell infiltration and promoting angiogenesis.

**Fig. 8:**
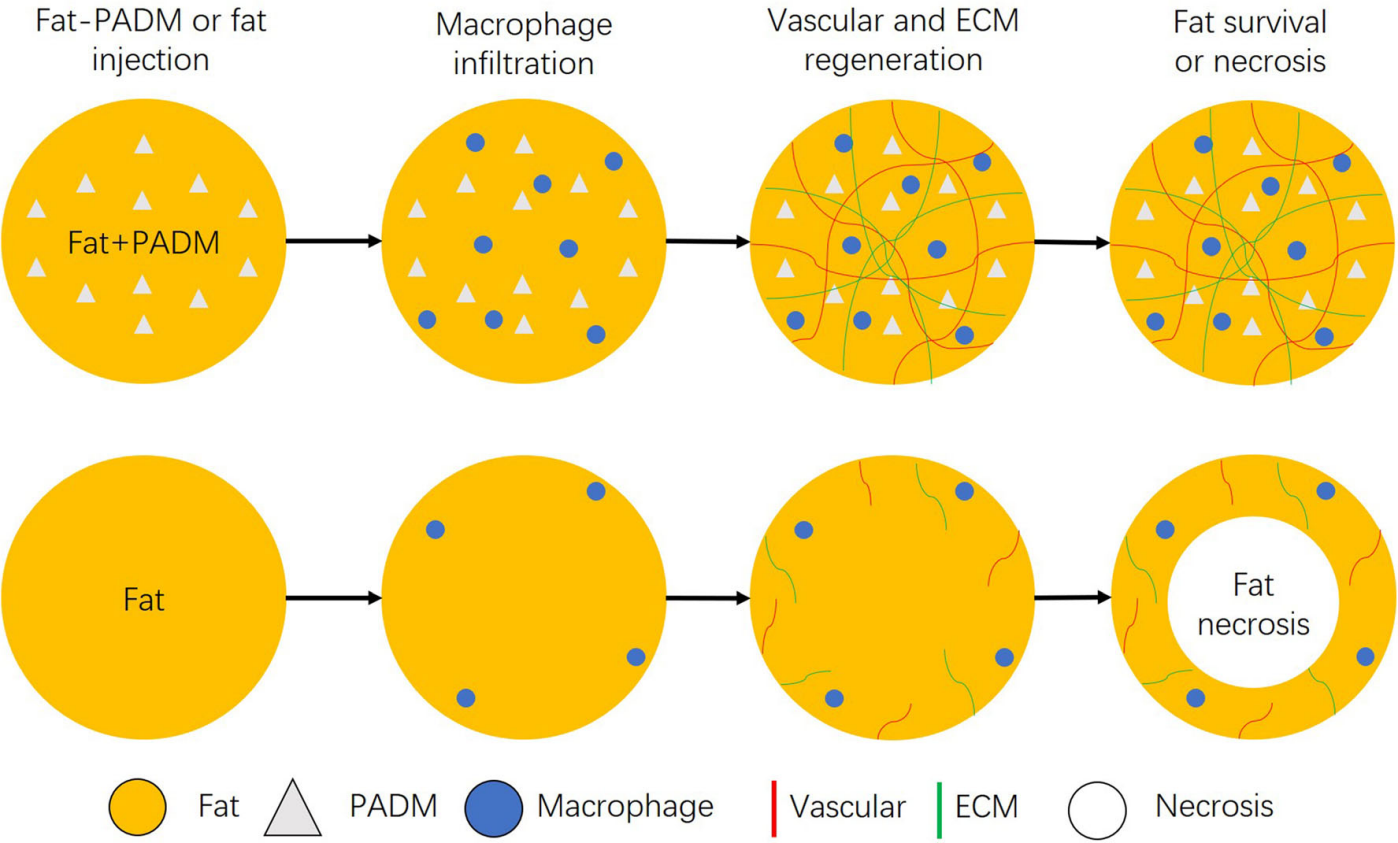
PADM increases fat survival rate after fat grafting

An exciting finding of our histological study was that PADM addition significantly promoted ECM regeneration. Later, RNA sequencing analysis showed that the regenerated ECM derived from mice. ECM is a collection of extracellular molecules, which not only supplies structural support for surrounding cells, but also regulates their migration, proliferation, and differentiation [27]. After grafting, the transplanted fat was exposed to oxidative and ischemic stress situations, and was surrounded by neutrophils and macrophages [28]. The inflammatory state significantly stimulates preadipocytes to synthesize a variety of ECM proteins [29]. Another study [30] found that liposuction can damage ECM’s structure. To heal as soon as possible, the transferred adipose tissue will reconstruct the ECM structure within one week. The early depletion of macrophages will decrease the expression of collagen I, collagen VI, and MMPs, and eventually hinder the ECM reconstruction. This means that after surgery, fat rapidly reconstructs ECM through inflammatory cells, providing more nutrients for fat survival, and promoting the opportunity for fat to communicate with surrounding tissues. Therefore, we posit that PADM supplies a good bridge for inflammatory cells and surrounding tissues to invade the transplanted fat, resulting in the ECM reconstruction (Fig. 8). All these cells supply an important proregenerative microenvironment for the survival of the adipocytes.

This study has some limitations. First, each mouse was injected with only 300 μl fat. The role of PADM in larger fat transplants stays unclear. Second, fat weight and liquefaction rate as survival indicators cannot stand for all cases, and future studies will have to use more evaluation methods. Third, it is still necessary to perfect the proportion of PADM in the mixture with fat. Fourth and last, this animal experiment confirmed that PADM is highly effective in improving fat survival. However, further clinical verification and long-term observations are needed to evaluate its safety and effectiveness.

## Conclusions

In conclusion, the present study proves that PADM is a good biomaterial that improves fat survival rate in fat grafting. This study has also shown a beneficial effect of added PADM on inflammatory cells infiltration and tissue regeneration. As a commercial product, PADM has good features conducive to its potential application in clinical autologous fat grafting.

## Declarations

### Human and animal participants

We performed all procedures and protocols of human and mice studies according to the recommendations of the National Institutes of Health Guidelines as approved by Shenzhen Second People’s Hospital (KS20190712004).
